# Elucidation of Spatial Distribution of Hydrophobic Aromatic Compounds Encapsulated in Polymer Micelles by Anomalous Small-Angle X-ray Scattering

**DOI:** 10.3390/polym10020180

**Published:** 2018-02-12

**Authors:** Shota Sasaki, Ginpei Machida, Ryosuke Nakanishi, Masaki Kinoshita, Isamu Akiba

**Affiliations:** Department of Chemistry and Biochemistry, The University of Kitakyushu, 1-1 Hibikino, Wakamatsu, Kitakyushu 8080135, Japan; x6maa006@eng.kitakyu-u.ac.jp (S.S.); w5maa014@eng.kitakyu-u.ac.jp (G.M.); w5maa003@eng.kitakyu-u.ac.jp (R.N.); w5maa008@eng.kitakyu-u.ac.jp (M.K.)

**Keywords:** anomalous small-angle X-ray scattering, polymer micelle, drug delivery system

## Abstract

Spatial distribution of bromobenzene (BrBz) and 4-bromophenol (BrPh) as hydrophobic aromatic compounds incorporated in polymer micelles with vesicular structure consisting of poly(ethylene glycol)-*b*-poly(*tert*-butyl methacrylate) (PEG-*b*-PtBMA) in aqueous solution is investigated by anomalous small-angle X-ray scattering (ASAXS) analyses near Br K edge. Small-angle X-ray scattering (SAXS) intensities from PEG-*b*-PtBMA micelles containing BrBz and BrPh were decreased as the energy of incident X-ray approached to Br K edge corresponding to the energy dependence of anomalous scattering factor of Br. The analysis for the energy dependence of SAXS profiles from the PEG-*b*-PtBMA micelles containing BrBz revealed that BrBz molecules were located in hydrophobic layer of PEG-*b*-PtBMA micelles. On the contrary, it was found by ASAXS that BrPh existed not only in the hydrophobic layer but also in the shell layer. Since ASAXS analysis successfully accomplished to visualize the spatial distribution of hydrophobic molecules in polymer micelles, it should be expected to be a powerful tool for characterization of drug delivery vehicles.

## 1. Introduction

When amphiphilic block copolymers are dissolved in aqueous solution, they undergo self-assembly into polymer micelles consisting of hydrophobic core and hydrated corona [1]. The polymer micelles have been expected to be drug carriers in drug delivery system (DDS) because they can uptake hydrophobic drug compounds in their hydrophobic cores in aqueous solution [2,3,4]. For the DDS particles, stable retention of drug compounds is the critical issue to reduce side effects [5]. The stability of retention of hydrophobic compounds should be related to their spatial distributions in polymer micelles. Therefore, it should be important to reveal the spatial distribution of hydrophobic compounds in polymer micelles.

In this study, we focus on anomalous small-angle X-ray scattering (ASAXS), which is small-angle X-ray scattering (SAXS) depending on the energy of incident X-ray near absorption edge of a targeted element, to reveal the spatial distribution of hydrophobic compounds incorporated in polymer micelles [6,7,8,9]. The ASAXS method has been applied for hard materials containing high atomic number elements because the K edges of high atomic number elements are generally located within the energy range of available synchrotron X-ray. Recently, the ASAXS method has also been applied for structural analyses of soft materials, such as micelles and polymer nanoparticles [9,10,11,12,13,14]. ASAXS corresponds to the variation of the atomic scattering factor *f* of a targeted element near its X-ray absorption edge. The energy dependence of *f* is described by the following equation.

(1) $$f\left(E\right)=f_{0}+f^{\prime }\left(E\right)+f^{\prime \prime }\left(E\right),$$

where *E* is the energy of incident X-ray, *f*_0_ is the normal atomic form factor and *f*’ and *f*” are the real and imaginary parts of anomalous scattering factor, respectively. Owing to the energy dependence of *f*’ and *f*”, X-ray scattering intensity shows energy dependence, which corresponds to scattering contribution of the targeted element, near the absorption edge. Therefore, analysis of the energy dependence of X-ray scattering intensity in small-angle region leads to the spatial distribution of targeted element. Thus, in this study, we apply the ASAXS method to reveal the spatial distribution of Br-labeled aromatic compounds incorporated in polymer micelles. Further, we discuss relation between molecular characteristics of aromatic compounds and their spatial distribution in polymer micelles.

## 2. Materials and Methods 

Reagents. Poly(ethylene glycol) methyl ether (4-cyano-4-pentanoate dodecyl trithiocarbonate) (*M*_n_ = 5.4 × 10^3^, *M*_w_/*M*_n_ = 1.1) as a chain transfer agent (CTA) was purchased from Sigma-Aldrich Co. Ltd. (St. Louis, MO, USA). *tert*-Butyl methacrylate (tBMA) was purchased from Tokyo Chemical Industry Inc. (Tokyo, Japan). 2,2′-Azobisisoputyronitrile (AIBN) was purchased from Kanto Chemicals Co. Ltd. (Tokyo, Japan). 4-Bromophenol (BrPh), bromobenzene (BrBz) and other solvents for syntheses were purchased from Wako Pure Chemicals Co. Ltd. (Tokyo, Japan). tBMA was purified by distillation under reduced pressure before polymerization. AIBN was recrystallized before it was used. The other reagents were used as obtained.

**Scheme 1:**
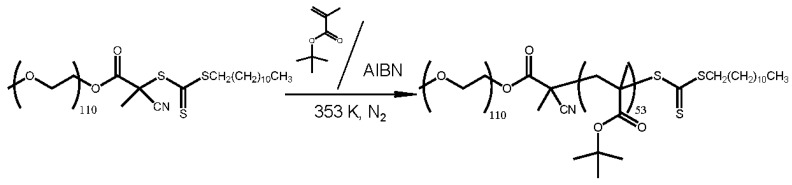
Synthesis of PEG-*b*-PtBMA by RAFT polymerization.

Synthesis of Poly(ethylene glycol)-*block*-poly(*tert*-butyl methacrylate) (PEG-*b*-PtBMA). Scheme 1 indicates the synthesis of PEG-*b*-PtBMA as an amphiphilic block copolymer. PEG-*b*-PtBMA was synthesized by reversible addition-fragmentation transfer (RAFT) radical polymerization. CTA (300 mg, 6.00 × 10^−2^ mmol) and AIBN (1.41 mg, 6.00 × 10^−3^ mmol) and *N*,*N*-dimethylformamide (DMF) (20 mL) were added into a flame-dried Schrenk Flask and purged with dry N_2_. Then, tBMA (853 mg, 6.00 mmol) was added to the flask with stirring under dry N_2_ atmosphere. After all reagents were dissolved in DMF, the flask was subjected to three freeze-pump-thaw cycles. RAFT polymerization was allowed to proceed for 20 h at 80 °C under dry N_2_ atmosphere and then quenched by immersion in liquid N_2_. PEG-*b*-PtBMA was isolated by precipitation from DMF solution to *n*-hexane. The number- and weight averaged molecular weight (*M*_n_ and *M*_w_) determined by ^1^H-NMR and size-exclusion chromatography (SEC) were 1.4 × 10^4^ and 1.7 × 10^4^, respectively. SEC elugrams and ^1^H-NMR spectrum of the resulting PEG-*b*-PtBMA were shown in Figures S1 and S2, respectively, in supplementary materials. ^1^H-NMR measurements for PEG-*b*-PtBMA in chloroform-d_1_ were performed by using a JEOL JNM ECP500 spectrometer. SEC measurements were performed by using a Shodex GPC K-804 column (eluent: chloroform, range of molar mass: 7000~300,000) combined with a JASCO RI-4030 differential refractive index detector and a JASCO PU-2087 HPLC pump at a flow rate of 1 mL·min^−1^.

Preparation of PEG-*b*-PtBMA micelles incorporating aromatic compounds. BrBz and BrPh were used as Br-labeled hydrophobic compounds. The PEG-*b*-PtBMA and BrPh or BrBz were mixed together at 10 wt % of aromatic compound against PEG-*b*-PtBMA. Micellization of the mixtures was induced by dissolving in tetrahydrofran (THF), stirring at an ambient temperature to ensure a homogeneous solution, followed by the gradual addition of 5 times the amount of ion exchanged water at a rate of 0.1 mL·min^−1^ via a syringe pump. The resulting solution was transferred to a dialysis tube (10 kDa MWCO) and dialyzed in a large amount of ion exchanged water for 3 days to remove THF. Finally, ion exchanged water was added to the micelle solution to a micelle concentration of 1.5 mg·mL^−1^. This concentration of hydrophobic compounds is the lower limit to obtain satisfactory data of anomalous small-angle X-ray scattering.

**Figure 1:**
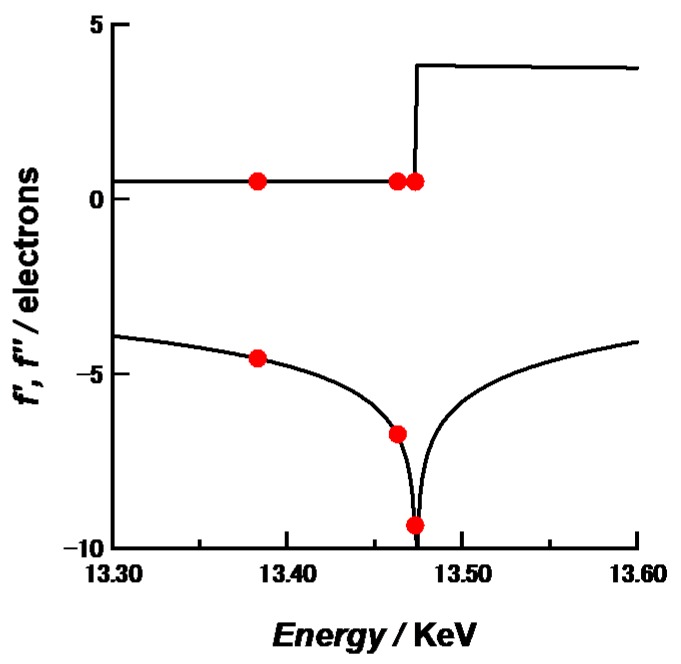
Energy dependences of *f*’ and *f*” of Br (solid lines) obtained by convolution of theoretical anomalous dispersions [15] and energy distribution in BL-40B2 station. Red circles indicate the energy of incident X-ray used in this study and corresponding *f*’ and *f*”.

Small-angle X-ray scattering (SAXS) and anomalous SAXS (ASAXS) measurements. SAXS and ASAXS measurements were performed at the BL-40B2 beamline of SPring-8, Japan. A 30 cm × 30 cm imaging plate (R-AXIS VII, Rigaku, Japan) was placed at a distance of 2 m from the sample position to cover a *q* range from 0.06 to 2.0 nm^−1^ at *λ* = 1.0 nm. Here, *q* = (4π/λ)sin(θ/2), where θ is the scattering angle and λ is the wavelength of the incident X-ray. A sample solution was packed in a quartz capillary with a light path length of 2.0 mm (Hilgenberg GmbH, Malsfeld, Germany). The X-ray transmittance of the samples was measured with ion chambers located in front of and behind the sample. The two-dimensional SAXS images obtained with an imaging plate were converted into one dimensional scattering intensity versus *q* profiles by circular averaging. To obtain excess scattering intensity *I*(*q*) at each *q*, scattering from the background were subtracted from the raw scattering data after an appropriate correction of transmittance. *I*(*q*) was corrected to the absolute scale using the absolute scattering intensity of water (1.632 × 10^−2^·cm^−1^) [9]. The exposure time of each SAXS measurement was kept at 1 min to avoid radiation damage and data were accumulated five times for each sample to obtain high signal-to-noise ratio. In addition, we have confirmed that SAXS profiles for just prepared samples agree with those for the sample aged for several weeks. Therefore, leakage of aromatic compounds and degradation have not occurred. Conventional SAXS measurements were carried out at an energy (wavelength) of incident X-ray of 12.40 keV (0.1 nm). For ASAXS measurements, three different energies 13.383, 13.463 and 13.473 keV were used as incident X-rays. Figure 1 shows the energy-dependence of the anomalous scattering factor of Br [9]. The K-edge of Br was determined as 13.483 keV. Because the three energies used here gave large differences of *f’* near the Br K-edge, they were selected as incident X-ray energies to obtain sufficient energy-dependence of scattering intensities for accurate ASAXS analyses. Table 1 summarizes the *f’* and *f”* values of Br at 13.383, 13.463 and 13.473 keV. The numerical analyses for SAXS and ASAXS data were carried out self-made programs on Igor 7 software.

Transmission electron microscope (TEM) observation. TEM was performed by using JEM-3010 (JEOL, Akishima shi, Japan). An accelerated voltage of 200 kV was used. The freeze-dried samples were placed on a carbon-spattered Cu grid and stained with Ti blue and lead acetate (II) to enhance TEM contrast.

## 3. Results and Discussion

**Figure 2:**
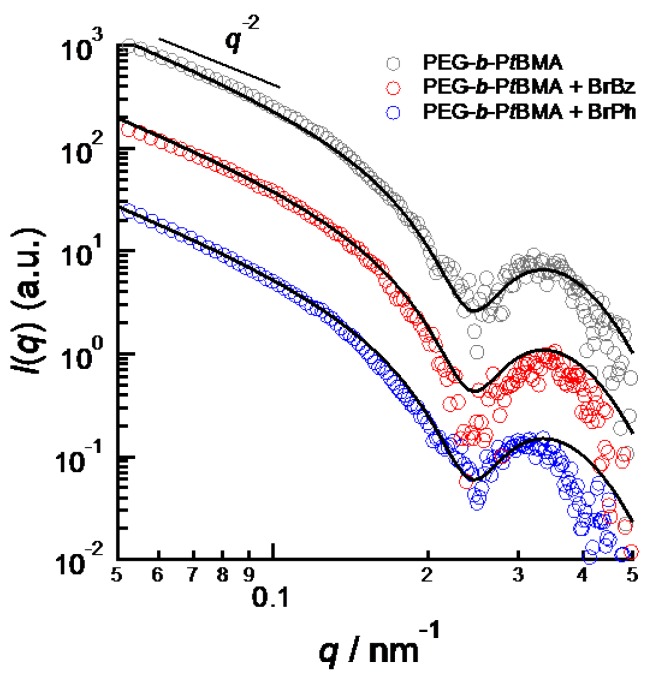
SAXS profiles from PEG-*b*-PtBMA micelles (gray), BrBz-containing PEG-*b*-PtBMA micelles (red) and BrPh-containing PEG-*b*-PtBMA micelles (blue).

**Figure 3:**
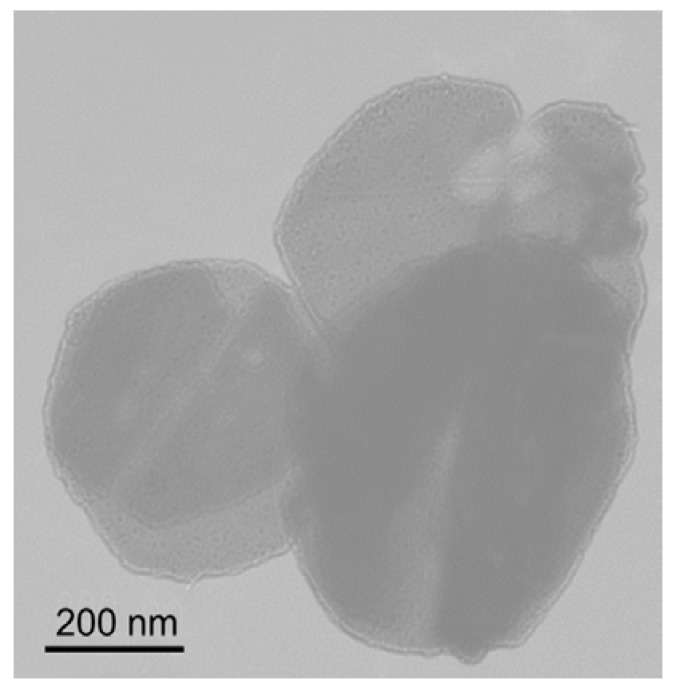
TEM micrograph of freeze-dried PEG-*b*-PtBMA micelles.

**Figure 4:**
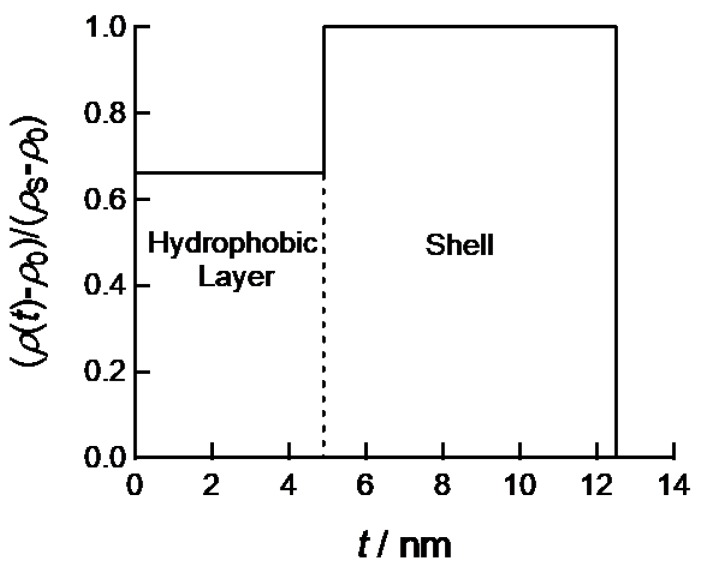
Cross-sectional electron density profile giving the best result in fitting analyses for SAXS profiles of PEG-*b*-PtBMA with and without Br-labeled compounds.

Figure 2 shows SAXS profiles of PEG-*b*-PtBMA micelles with and without aromatic compounds. Because of dilute concentration of PEG-*b*-PtBMA micelles, the SAXS profiles are regarded as form factors of micelles. The SAXS intensity of PEG-*b*-PtBMA micelle in low *q* region shows *q*^−2^ dependence. The *q*^−2^ dependence of SAXS intensity indicates that PEG-*b*-PtBMA micelles take disk-like or vesicular forms. TEM photo for freeze-dried PEG-*b*-PtBMA micelles shown in Figure 3 reveals that the PEG-*b*-PtBMA forms vesicular micelles. The SAXS profiles of PEG-*b*-PtBMA micelles containing BrBz and NrPh also show the *q*^−2^ dependence in low *q* regions. Therefore, the vesicular structure of PEG-*b*-PtBMA micelles is maintained by incorporating aromatic compounds in their inside. Although the radius of PEG-b-PtBMA micelles cannot be estimated by SAXS because of their extremely large size as shown in TEM photo, the thickness of the membrane of PEG-*b*-PtBMA micelles can be estimated. The solid lines in Figure 2 show the same theoretical SAXS curves calculated for a core-shell platelet model as described by the following equation [16]:

(2) $$I\left(q\right)\propto \left[\frac{4\pi }{q^{4}}{\left\{\left(\rho_{C}-\rho_{S}\right)\sin\left(q\frac{t_{C}}{2}\right)+\left(\rho_{S}-\rho_{0}\right)\sin\left[q\left(\frac{t_{S}}{2}+t_{S}\right)\right]\right\}}^{2}\right],$$

where *t*_C_ and *t*_S_ are the half thicknesses of a core and overall of a plate, respectively and *ρ*_C_, *ρ*_S_ and *ρ*_0_ are the electron densities of core, shell and solvent, respectively. By assuming the cross-sectional electron density profile as shown in Figure 4, the calculated SAXS curve can be fitted to experimental SAXS profiles of PEG-*b*-PtBMA micelles with or without aromatic compounds. The half-thickness of hydrophobic core layer and thickness of shell layer are estimated to 4.7 and 7.8 nm, respectively. As shown in Figure 2, the scattering curve calculated by the cross-sectional electron density profile shown in Figure 4 can describe all SAXS data of PEG-*b*-PtBMA micelles and the micelles with BrBz and BrPh. These thicknesses well agree with the radii of gyration estimated by molecular weights of PtBMA and PEG chains. Hence, PEG-*b*-PtBMA micelles are consisting of single core layer sandwiched by hydrophilic shell layers. Therefore, effect of addition of small amounts of hydrophobic compounds on structure of polymer micelles can be ignored. For the PEG-*b*-PtBMA micelles incorporating BrBz and BrPh, ASAXS measurements near Br K edge are applied to figure out the spatial distribution of BrBz and BrPh in the micelles.

**Figure 5:**
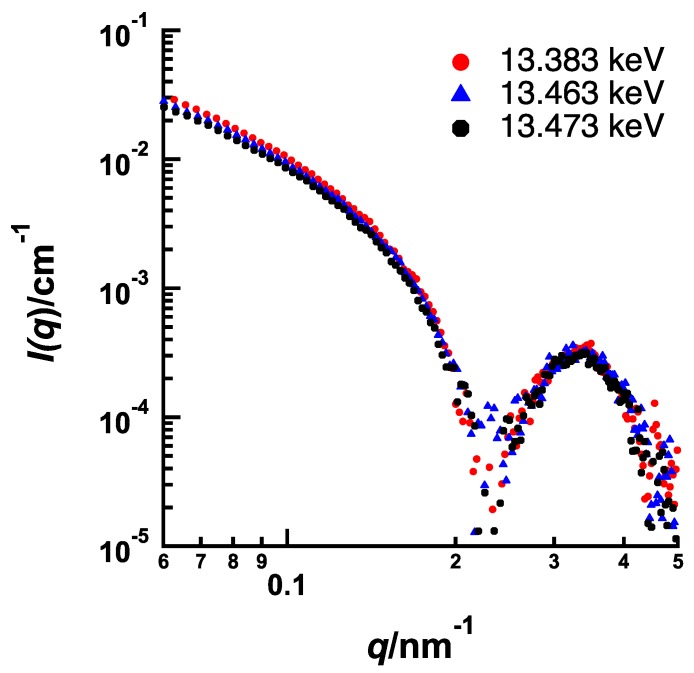
Energy dependence of SAXS profiles of PEG-*b*-PtBMA containing BrBz near Br K edge (13.483 keV).

**Figure 6:**
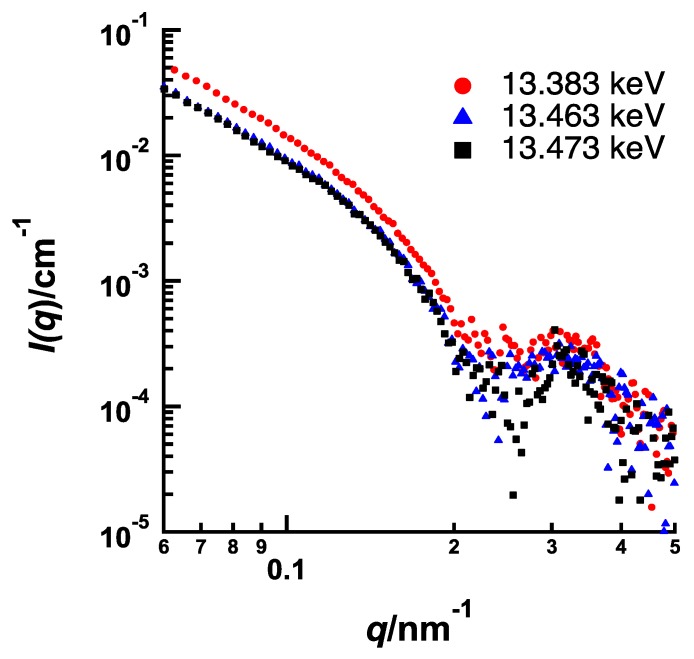
Energy dependence of SAXS profiles of PEG-*b*-PtBMA containing BrPh near Br K edge (13.483 keV).

Figure 5 and Figure 6 show the energy dependences of SAXS profile from BrBz- and BrPh-containing PEG-*b*-PtBMA micelles, respectively, measured at 13.383 keV (0.927 nm), 13.463 keV (0.921 nm) and 13.473 keV (0.920 nm). In both systems, SAXS intensities are decreasing as the energy of incident X-ray approaches from lower energy to Br K edge, although the features of energy resonance are different. The energy dependence of SAXS intensity originated from that of the *f*’ of Br as shown in Figure 1 is described by the following equation.

(3) $$I\left(q,E\right)=N\left\{P_{0}\left(q\right)+2f^{\prime }\left(E\right)A\left(q\right)V\left(q\right)+\left({f^{\prime }}^{2}\left(E\right)+{f^{\prime \prime }}^{2}\left(E\right)\right)V^{2}\left(q\right)\right\},$$

where *P*_0_(*q*) is the non-resonant (normal) form factor of micelle and *V*^2^(*q*) is the form factor of resonant part. In this case, the *V*^2^(*q*) corresponds to the form factor of the area where Br atoms are existing. Consequently, difference of *V*^2^(*q*) profiles causes the difference of features of energy resonance in SAXS profiles. Therefore, it is strongly suggested that the spatial distribution of BrBz in PEG-b-PtBMA micelles is much different from that of BrPh. By dissolving simultaneous equation of the SAXS profiles measured at three different energies of incident X-ray, we can obtain *V*^2^(*q*) profile as described by the following equation.

(4) $$V^{2}\left(q\right)=\frac{1}{K}\left[\frac{\Delta I\left(q,\;E_{1},\;E_{2}\right)}{{f^{\prime }}_{Br}\left(q,\;E_{1}\right)-{f^{\prime }}_{Br}\left(q,\;E_{2}\right)}-\frac{\Delta I\left(q,\;E_{1},\;E_{3}\right)}{{f^{\prime }}_{Br}\left(q,\;E_{1}\right)-{f^{\prime }}_{Br}\left(q,\;E_{3}\right)}\right],$$

where

(5) $$K={f^{\prime }}_{Br}\left(E_{2}\right)-{f^{\prime }}_{Br}\left(E_{3}\right)+\frac{f{\prime \prime }_{Br}{}^{2}\left(E_{1}\right)-f{\prime \prime }_{Br}{}^{2}\left(E_{2}\right)}{{f^{\prime }}_{Br}\left(E_{1}\right)-{f^{\prime }}_{Br}\left(E_{2}\right)}-\frac{f{\prime \prime }_{Br}{}^{2}\left(E_{1}\right)-f{\prime \prime }_{Br}{}^{2}\left(E_{3}\right)}{{f^{\prime }}_{Br}\left(E_{1}\right)-{f^{\prime }}_{Br}\left(E_{3}\right)},$$

**Figure 7:**
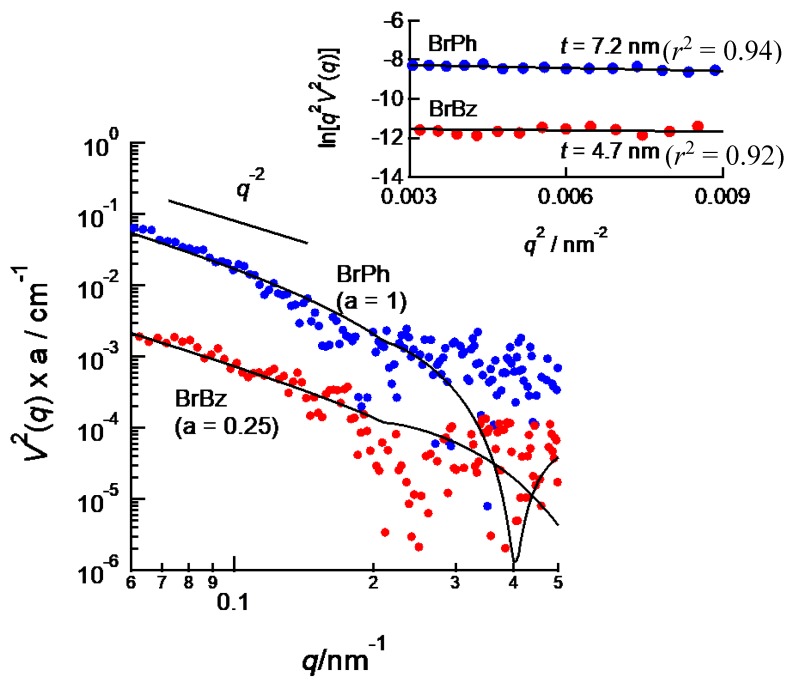
*V*^2^(*q*) profiles of BrBz- (red) and BrPh-containing (blue) PEG-*b*-PtBMA micelles. Solid lines are calculated by theoretical scattering functions for disk-like particles with 4.7 and 7.2 nm of half-thickness estimated from thickness Guinier plots. Insert shows thickness Guinier plot of *V*^2^(*q*) of BrBz- and BrPh-containing PEG-*b*-PtBMA micelles. The half-thickness of BrBz and BrPh areas are estimated to 4.7 and 7.2 nm, respectively.

Figure 7 shows *V*^2^(*q*) profile of BrBz-containing and BrPh-containing PEG-*b*-PtBMA micelles. Because of low resolution of ASAXS treatment in high *q* region, detail analysis for *V*^2^(*q*) by using scattering function of model particle is difficult. However, both *V*^2^(*q*) profiles obviously show *q*^−2^ dependences in low *q* region. This means the area where Br-labeled aromatic compounds are distributed takes a platelet form. Therefore, the vesicular structure of PEG-*b*-PtBMA micelle is reflected to the spatial distribution of BrBz and BrPh, like templates. For platelet particles like a membrane of vesicle, relation between scattering intensity *I*(*q*) (or resonant scattering term *V*^2^(*q*)) and *q* in low *q* region is given by the following equation.

(6) $$q^{2}I\left(q\right)\propto \;\exp\left(-t^{2}q^{2}\right),$$

where *t* is the half-thickness of a platelet particle. Therefore, the slope of plots of *q*^2^*V*^2^(*q*) against *q*^2^—so-called thickness Guinier plots—half-thicknesses of platelet particles can be estimated. From the thickness Guinier plots as shown in the insert of Figure 7, the half-thicknesses of the platelet areas are estimated to 4.7 and 7.2 nm for BrBz and BrPh, respectively. As shown in the insert of Figure 7, the thicknesses estimated in low *q* region are obtained with sufficient accuracy, although the SAXS data in high *q* region include large uncertainty. By using these results, scattering curves for *V*^2^(*q*) profiles for BrBz- and NrPh-containing micelles are calculated by using disk-like particles. The solid lines in Figure 7 are calculated curves. Although the *V*^2^(*q*) profiles in high *q* region contain large noise, the calculated curves agree with *V*^2^(*q*) profiles in low q region. The thickness of BrBz area is close to that of hydrophobic layer of PEG-*b*-PtBMA micelles shown in Figure 4. Therefore, it should be considered that BrBz molecules are homogeneously dispersed in hydrophobic layer of PEG-*b*-PtBMA micelles. On the contrary, the half-thickness of BrPh area is much larger than that of hydrophobic layer of PEG-*b*-PtBMA micelle. This result means that a part of BrPh filtrates to hydrated shell layer of PEG-*b*-PtBMA micelles. This result is consistent with the spatial distribution of a phenol derivative incorporated in spherical polymer micelles reported by Sanada et al. [14] and some of our previous studies [13,17]. BrPh can form hydrogen bonds with both PtBMA and PEG. In addition, the number density of PEG chains near core-shell interface is much higher than in the outer region of shell. Consequently, BrPh can exist in shell layer composed of PEG near the core-shell interface. According to the result in this study, it is strongly suggested that spatial distribution of hydrophobic compounds incorporated in polymer micelles can be controlled by tuning interactions not only between hydrophobic compounds and not only hydrophobic polymers but also water-soluble polymers of amphiphilic block copolymers.

## 4. Conclusions

ASAXS near Br K edge was applied for analyses of the spatial distribution of hydrophobic aromatic compounds (BrBz and BrPh) encapsulated in polymer micelles consisting of PEG-*b*-PtBMA. It was found that BrBz was dispersed in hydrophobic layer of PEG-*b*-PtBMA micelles, while BrPh was infiltrated to hydrophilic shell layer. This result inevitably leads the conclusion that the spatial distribution of hydrophobic compounds encapsulated in polymer micelles can be controlled by tuning intermolecular interactions, such as hydrogen bonds, between hydrophobic compounds and each chain of amphiphilic block copolymers.

## Figures and Tables

**Table 1:** Values of real and imaginary parts of anomalous scattering factors (*f’* and *f”*) at 13.383, 13.463 and 13.473 keV.

Energy/keV	*f’*	*f”*
13.383	−4.56	0.508
13.463	−6.76	0.503
13.473	−9.33	0.502

## Supplementary Materials

The following are available online at http://www.mdpi.com/2073-4360/10/2/180/s1, Figure S1: GPC elugrams of PEG (blue) and PEG-*b*-PtBMA (Red), Figure S2: ^1^H-NMR spectrum of PEG-*b*-PtBMA in CDCl_3_.

## References

[1] Zhang L.F., Eisenberg A. (1995). Multiple Morphologies of “Crew-Cut” Aggregates of Polysyrene-*b*-poly(acrylic acid) Block Copolymers. Science.

[2] Yokoyama M., Kwon G.S. (2005). Polymeric Micelles for the Targeting of Hydrophibic Drugs. Polymeric Drug Delivery Systems.

[3] Monfardini C., Veronese F.M. (1998). Stabilization of Substances in Circulation. Bioconjugate Chem..

[4] Matsumura Y., Kimura M., Yamamoto T., Maeda H. (1988). Involvement of the Kinin-generating Cascade in Enhanced Vascular Permeability in Tumor Tissue. Jpn. J. Cancer Res..

[5] Yamamoto T., Yokoyama M., Opanasopit P., Hayama A., Kawano K., Maitani Y. (2007). What Are Determining Factors for Stable Drug Incorporation into Polymeric Micelle Carriers? Consideration on Physical and Chemical Characters of the Micelle Inner Core. J. Control. Release.

[6] Materlik G., Sparks C.J., Fischer K. (1994). Resonant Anomalous X-ray Scattering: Theory and Applications.

[7] Waseda Y. (2002). Anomalous X-ray Scattering for Materials Characterization: Atomic-Scale Structure Determination.

[8] Stuhrmann H.B. (1985). Resonance scattering in macromolecular structure research. Adv. Polym. Sci..

[9] Akiba I., Takechi A., Sakou M., Handa M., Shinohara Y., Amemiya Y., Yagi N., Sakurai K. (2012). Anomalous Small-Angle X-Ray Scattering Study of Structure of Polymer Micelles Having Bromines in Hydrophobic Core. Macromolecules.

[10] Patel M., Rosenfeldt S., Ballauff M., Dingenouts N., Pontoni D., Narayanan T. (2004). Analysis of the Correlation of Counterions to Rod-like Macroions by Anomalous Small-angle X-ray Scattering. Phys. Chem. Chem. Phys..

[11] Sztucki M., Cola E.D., Narayanan T. (2011). New Opportunities for Anomalous Small-Angle X-Ray Scattering to Characterize Charged Soft Matters Systems. J. Phys. Conf. Ser..

[12] Sakou M., Takechi A., Murakami S., Sakurai K., Akiba I. (2013). Study on the Internal Structure of Polymer Micelles by Anomalous Small-angle X-ray Scattering at Two Edges. J. Appl. Cryst..

[13] Sanada Y., Akiba I., Sakurai K., Shiraishi K., Yokoyama M., Mylonas E., Ohta N., Yagi N., Shinohara Y., Amemiya Y. (2013). Hydrophobic Molecules Infiltrating into the Poly(ethylene glycol) Domain of the Core/Shell Interface of a Polymeric Micelle: Evidence Obtained with Anomalous Small-Angle X-ray Scattering. J. Am. Chem. Soc..

[14] Nakanishi R., Machida G., Kinoshita M., Sakurai K., Akiba I. (2016). Anomalous Small-angle X-ray Scattering Study on the Spatial Distribution of Hydrophobic Molecules in Polymer Micelles. Polym. J..

[15] Sasaki S. (1989). Anomalous Scattering Factors for Synchrotron Radiation Users, Calculated Using Cromer and Liberman’s Method.

[16] Pons R., Valiente M., Montalvo G. (2010). Structure of Aggregates in Diluted Aqueous Octyl Glucoside/Tetraethylene Glycol Monododecyl Ether Mixtures with Different Alkanols. Langmuir.

[17] Nakanishi R., Kinoshita M., Sasaki S., Akiba I. (2016). Spatial Distribution of Hydrophobic Compounds in Polymer Micelles as Explored by Anomalous Small-angle X-ray Scattering near Br K-edge. Eur. Polym. J..

