# Supplementary material for: Elucidation of Spatial Distribution of Hydrophobic Aromatic Compounds Encapsulated in Polymer Micelles by Anomalous Small-Angle X-ray Scattering
Source: Polymers (Basel). 2018 Feb 12;10(2):180. doi: 10.3390/polym10020180 (PMC6415145; doi:10.3390/polym10020180)
Supplement: Supplementary file 1 [file polymers-10-00180-s001.pdf]

Supplementary materials for

Elucidation of Spatial Distribution of Hydrophobic Aromatic Compounds Encapsulated in Polymer Micelles by Anomalous Small-angle X-ray Scattering

*Shota Sasaki, Ginpei Machida, Ryosuke Nakanishi, Masaki Kinoshita, and Isamu Akiba**

GPC elugrams and ^{1}H-NMR spectrum for PEG-*b*-PtBMA.

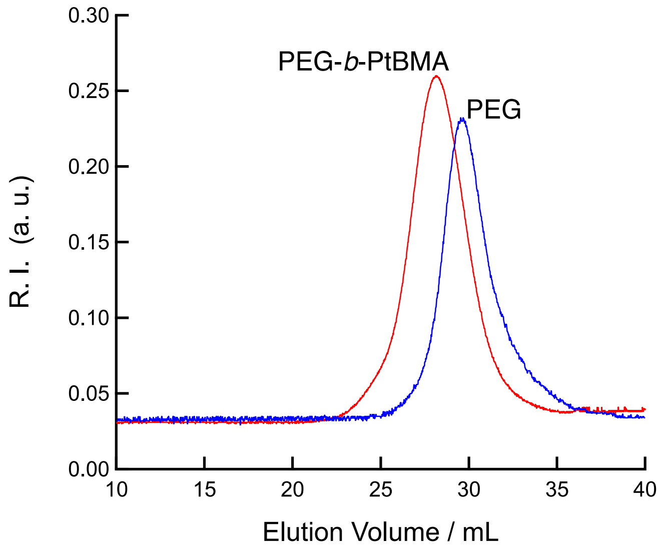

Figure S1. GPC elugrams of PEG (blue) and PEG-*b*-PtBMA (Red).

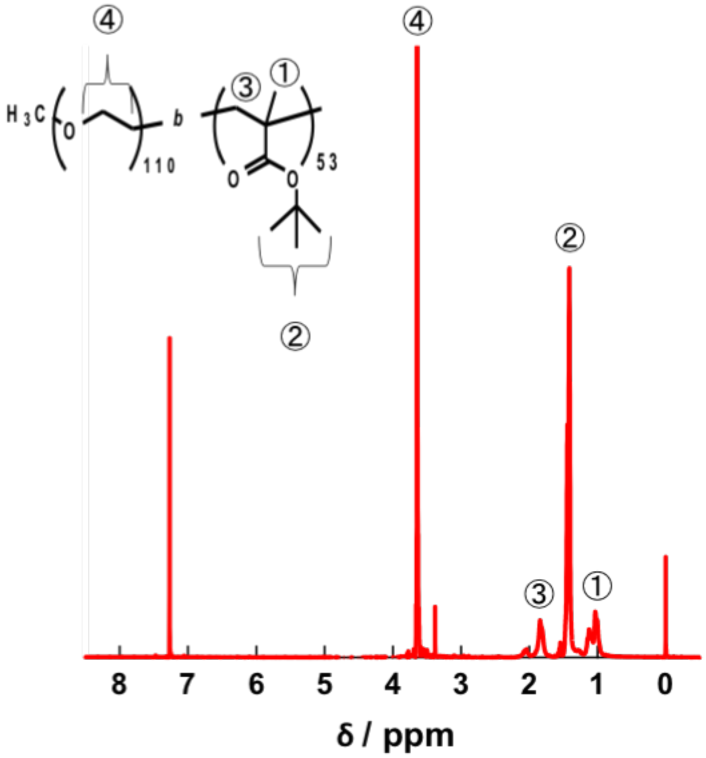

Figure S2. ^{1}H-NMR spectrum of PEG-*b*-PtBMA in $CDCl_3$.
